# Influence of age and level of activity on the applicability of a walker orthosis - a prospective study in different cohorts of healthy volunteers

**DOI:** 10.1186/s12891-018-2366-2

**Published:** 2018-12-22

**Authors:** Alena Sint, Sebastian Felix Baumbach, Wolfgang Böcker, Christian Kammerlander, Karl-Georg Kanz, Mareen Braunstein, Hans Polzer

**Affiliations:** 1 Department of General-, Visceral-, Vascular- and Transplant- Surgery, Munich University Hospital, Ludwig-Maximilians-University (LMU), Munich, Germany; 2 Department of General, Trauma and Reconstructive Surgery, Munich University Hospital, Ludwig-Maximilians-University (LMU), Nussbaumstr 20, 80336 Munich, Germany; 3 Department of Trauma Surgery, Klinikum rechts der Isar, Technical University of Munich, Munich, Germany

**Keywords:** Orthosis, Ankle immobilisation, Level of activity, Elderly, Patient safety

## Abstract

**Background:**

Walker orthosis are frequently prescribed as they are removable to allow wound control, body care and physiotherapy and are adaptable to the soft tissue conditions. The prerequisite for successful treatment with any walker orthosis is a correct use by the patient. Therefore, the aim of this study was to investigate patients’ handling of a commonly used walker.

**Methods:**

Prospective observational study analyzing the applicability of a walker orthosis in different cohorts with varying age and level of activity. Volunteers were recruited from a mountain-biking-team (Sport), a cardiovascular-health-sports-group (Cardio) and a retirement home (Senior). The correct application was assessed following initial training (t0) and one week later (t1). Outcome parameters were an Application Score, strap tightness, vertical heel lift-off and subjective judgement of correct application.

**Results:**

Thirty-three volunteers, 11 Sports group (31 ± 7a), 12 Cardio group (59 ± 11a), 10 Senior group, (82 ± 5a) were enrolled. No differences for any parameter could be observed between t0 and t1. Age showed a moderate correlation for all outcome parameters and the cohort influenced all variables. The Senior group presented significant inferior results to the Sport- and Cardio group for the Application Score (*p* = 0.002-*p* < 0.001) and strap tightness (*p* < 0.001). Heel lift-off was significantly inferior in the Cardio- and Senior- compared to the Sport group (*p* = 0.003-*p* < 0.001). 14% in the Sport-, 4% in the Cardio- and 83% in the Senior group achieved less than 9 points in the Application Score – which was considered insufficient. However, out of these 90% believed the application to be correct.

**Conclusions:**

The elderly cohort living in a retirement home demonstrated an impaired handling of the walker orthosis. Further, participants were incapable to self-assess the correct handling. These aspects should be respected when initiating treatment with a walker orthosis.

**Trial registration:**

Retrospectively registered on the 16th of February 2018: #DRKS00013728 on DRKS.

**Electronic supplementary material:**

The online version of this article (10.1186/s12891-018-2366-2) contains supplementary material, which is available to authorized users.

## Background

Various pathologies affecting the foot and ankle require immobilization. These conditions include severe sprains, Achilles tendon ruptures, fractures, after care after arthrodesis and many more. The proportion of elderly patients necessitating ankle immobilization is notable. As an example more than 80% of all foot and ankle fractures occur in women aged 75 years or above [1]. These figures are expected to increase within the next 20 years [1, 2]. A similar trend can be observed for ankle (55 ± 12 years) and hindfoot (58 ± 16 years) arthrodesis [3]. Consequently, the proportion of elderly patients necessitating ankle immobilization is considerable and will further increase. Immobilization can be achieved by various means, including casts, splints or walker orthosis. Over the last decade, walkers have become increasingly popular [4], although up to date no independent and verifiable data is available in regard to the exact frequency. The reason is that walkers feature several advantages: First, they are removable and therefore allow early physiotherapy to prevent muscular atrophy [5] and arthrofibrosis [6]. Second, they allow easy wound control and body care [6, 7]. Finally, walker orthosis are adjustable and can be adapted to the current soft tissue conditions [7]. Therefore, walker orthosis are becoming more and more popular also for treatment of diabetic foot ulcerations as an alternative to total contact casts [8, 9], as they relieve pressure under the forefoot [10].

Today, various walker orthosis are available. In general one can distinguish short orthosis extending just proximal of the ankle joint which do not do not provide immobilization of the ankle from those extending just below the knee joint. These longer orthosis seem much more frequently applied e.g. in ankle fracture treatment or for plantar ulceration as they immobilize the ankle and relieve pressure under the forefoot [10]. Their design is comparable, consisting of a stable lower plastic shell with a sole, a fabric liner and a removable upper shell, which is fixed to the lower shell by straps. The prerequisites for any walker orthosis is an easy and safe handling to ensure sufficient immobilization as the walker is removed and reapplied by the patient. Therefore, the handling must be intuitive and reproducible. In typical clinical practice the orthosis is applied to the patients’ lower leg by a trained nurse / physiotherapist. During this procedure the basic tasks of the application are explained. Thereafter, the patient is handed the manufactures instructions and will be self-dependent for handling the orthosis. Especially elderly patients might be hindered due to cognitive impairment, sarcopenia or limited flexibility. The authors are not aware of a study investigating patient safety for any walker orthosis.

## Methods

### Aim and study design

The aim of this study was to assess patient handling safety of a commonly prescribed walker orthosis in a broad sample. The design was a prospective study analysing the applicability of a walker orthosis in different cohorts with varying age and level of activity. The study was approved by the local ethics committee (#782–16).

### Population

Thirty-three healthy individuals from different cohorts varying in age and the level of activity were recruited. To assure different levels of activity, individuals from three different cohorts were recruited: young volunteers from a mountain biking team (Sport), independently living elderly from a cardiovascular health sports group (Cardio) and elderly living in a retirement home (Senior). The inclusion criteria were no musculoskeletal impairment within the last six months, age between 18 and 95 years and informed consent. Exclusion criteria were distinct cognitive, neurological or obvious physical impairment, acute impairment of the foot and ankle within the last 6 months, pregnancy, or inability to give informed consent. Cognitive abilities were implied as all participants were living self-dependant, organizing their everyday live without assistance. Also the participants living in a retirement home were not dependent on fostering but were living in autonomous apartments receiving assistance only in the case of emergency. Neurological and obvious physical impairment were evaluated by assessment of the patients’ medical history as well as assessment of their capabilities during the appointments in the course of this study.

### General aspects of the walker orthosis

**Fig. 1:**
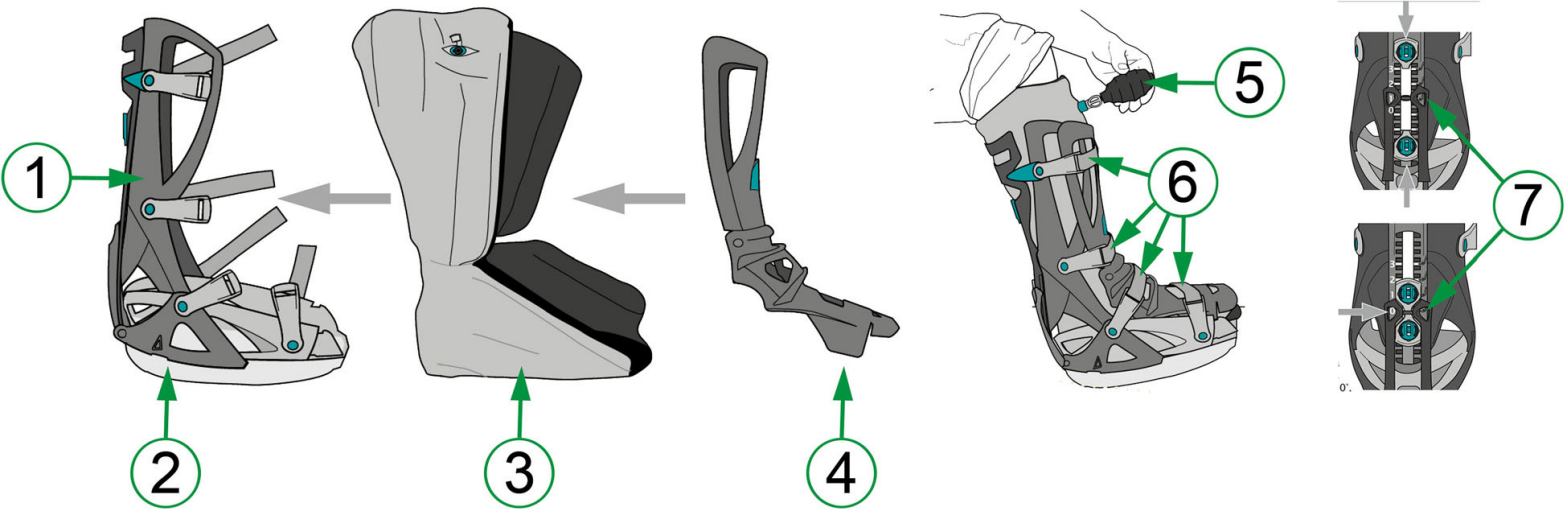
Schematic illustration of the VACOPed®. 1. Lower shell; 2. Durable sole; 3. Vacuum cushion in fabric liner; 4. Upper shell; 5. Bulb pump; 6. Adjustable belt straps; 7. Adjustable joint

The walker used in this study was the VACO®ped (P2, OPED GmbH, Valley, Germany). It is a patient operated, modular walker (Fig. 1), consisting of a lower plastic shell (Fig. 1.1), a detachable sole (Fig. 1.2), a vacuum cushion in fabric liner (Fig. 1.3) and a removable upper shell (Fig. 1.4). A vacuum pump (Fig. 1.5) is used to make the vacuum cushion rigid. The upper shell is secured to the lower shell by four adjustable belt straps (Fig. 1.6). Ankle range of motion is adjustable (Fig. 1.7).

### Participant instructions

**Fig. 2:**
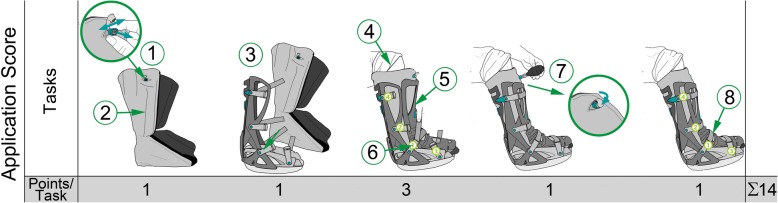
Schematic illustration of the Application score. 1. Pulling the valve of the vacuum cushion to allow air to enter; 2. Fluffing the vacuum cushion; 3. Placement of the foot in the vacuum cushion and adjustment of the vacuum cushion in the lower shell; 4. Pushing the heel into the vacuum cushion; 5. Placement of the upper shell over the front of the vacuum cushion; 6. Locking the straps in correct sequence (starting with the inner straps 1 and 2 and completing with the outer straps 3 and 4); 7. Making the vacuum cushion rigid using the bulb pump; 8. Fastening all four straps. The number of points assigned to each task is depicted in the lower row

The instruction session was designed to be on the one hand comparable to the everyday clinical situation and on the other hand standardized and reproducible from a scientific perspective. Therefore, the instruction session was standardized including a demonstration and a repetition section. The instruction session was performed by a single person specially trained in the use of the orthosis and this standardized protocol. The participants were trained in small groups with a maximum of four people. The standardized instruction session is depicted in the following: First, the instructor explained the application using a walker orthosis as demonstration material. The instructions followed the tasks depicted in the Application Score (Fig. 2). Thereafter, the instructor applied the walker orthosis to the own ankle, again recapitulating the instructions step by step. Afterwards, each participant applied the orthosis by himself under supervision of the instructor. The participants were instructed to tighten the straps as tight as possible without causing pain or impairment of sensitivity to their foot or ankle. Finally, all remaining questions were answered, and the patients were handed the instruction leaflet provided by the manufacturer along with the orthosis.

### Data collected

Following thorough training on the handling of the orthosis, participants self-applied the walker orthosis and the proper fit was assessed according the parameters outlined below (t0). The data collected are depicted in Table 1. Next to general demographics, the correct application of the walker was assessed by the 8-point Application Score (Fig. 2). The proper immobilization was objectified by strap tightness and heel mobility. Finally, subjective judgement was evaluated. Application reproducibility was assessed by the same set of tasks repeated one week later without instructions (t1). Data acquisition during the both testing sessions (T0 / t1) took place with only a single participant one by one in a separate room.

**Table 1:** Outline of the data assessed

General demographics	Age	
	Sex	
	Shoe size	
	Cohort: Sport, Cardio, Senior	
Time	t0	t1
Quantitative Assessment	Application Score	
	Strap tightness	
	Heal lift-off	
Subjective Judgement	Proper application (right/wrong)	

*t0* Initial assessment, *t1* Repeated measurements one week later

#### Application score

In order to objectify the correct application of the walker orthosis the authors developed an 8-scale, non-linear Application Score (Fig. 2). The application process of the walker was divided into eight tasks. As the individual tasks are of varying importance for the functionality of the orthosis, they were weighted accordingly. The minor tasks (1, 2, 3, 7 and 8) accounted for one point each as they are of minor importance for the clinical effectiveness of the orthosis. On the other hand, positioning of the foot within the walker (task 4), placement of the upper shell (task 5), and locking of the straps (task 6) are of greater importance and therefore were considered major tasks accounting for three points each. The Application Score and the points appointed to each task are depicted in Fig. 2. The minimum score was 0 points, the maximum 14 points. In order to assure the clinical effectiveness of the walker, at least two of the three major tasks (number 4 to 6) had to be completed correctly. If two major tasks were not completed correctly, the application of the orthosis was considered insufficient. Consequently, an Application Score of less than 9 points resembled an insufficient application of the orthosis. On the other hand an Application Score of ≥9 points was considered sufficient.

#### Strap tightness

**Fig. 3:**
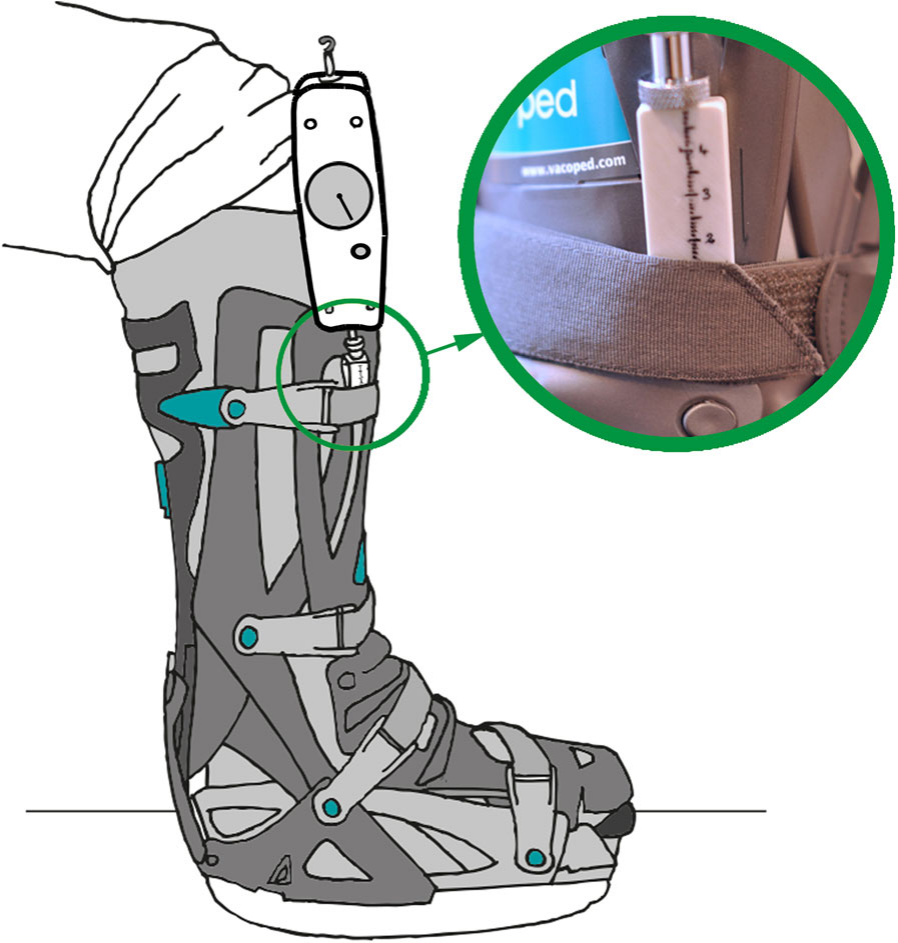
Schematic illustration of the measurement for strap tightness

Strap tightness was assessed as the penetration depth of a wedge in centimetres at a constant force of 20 N. Therefore, a scaled, rigid plastic wedge was attached to a pressure force gauge (Analog Push Pull Gauge SN-50, Sundoo Instruments, Zhejiang, China). The wedge was inserted between each strap and the upper shell at standardized marked positions (Fig. 3). The penetration depth at 20 N was noted for each strap. A low penetration depth of the wedge corresponded to a strong strap tightness and vice versa. Norm values for adequate strap tightness were generated prior to study initiation. Therefore, a Reference group of eleven employees of the manufacturer, with a special expertise in handling the walker orthosis, were asked to apply the walker. Strap tightness was then assessed as outlined above. Norm values were defined as the average value of the eleven experts. Strap tightness was analysed pooled and for each strap separately.

#### Heel lift-off

The proper immobilization of the foot and ankle was further quantified by assessing the vertical heel motion within the orthosis. The volunteers were asked to lift the heel as far as possible within the orthosis. The distance between the heel and the bottom of the orthosis was assessed by inserting blocks in 0.5 cm increments underneath the heel through a slit at the dorso-lateral aspect of the cushion without disassembling the orthosis. Norm values for heel lift-off were also generated from the Reference group prior to the study.

#### Subjective judgement

The volunteers were asked to subjectively judge, whether the walker was applied correctly (right/false).

### Outcome parameters

The primary outcome parameter was the Application Score. The secondary outcome parameters were strap tightness, heel-lift off, and the subjective judgement. Subgroup analysis aimed at assessing the reproducibility of the outcome parameters (t0 vs. t1) as well as a possible influence of gender, age and the cohort from which the subjects were recruited. The subjective judgement was compared to the Application Score per its clinical effectiveness (Application Score values < 9 ≥ points).

### Statistical analyses

Due to missing preliminary data, no sample size estimation could be conducted. The Shapiro-Wilk Test revealed a normal distribution. All values in the following are stated as mean values ± standard deviation (range). Next to standard descriptive statistics, paired and independent t-tests, Pearson-Correlations, and ANOVA (post hoc Bonferroni) were conducted where appropriate. The level of significance was set at *p* = 0.05. Statistics were computed using SPSS Vs. 21 (IBM Company).

## Results

### Patient population

Thirty-three volunteers, 11 in the Sports- (31 ± 7 years), 12 in the Cardio- (59 ± 11 years) and 10 in the Senior group (82 ± 5 years) were enrolled. 49% were female, the mean shoe size (EU) was 41 ± 3 (37–46) and 15/18 used a S/M sized VACO®ped. Demographics per cohort are presented in Table 2. Five subjects were excluded at follow-up (t1) for the following reasons: absence without leave (*n* = 1, 37 years, Sport), incapability to close the straps due to acute physical impairment (*n* = 2, 77/85 years, Senior), totally incorrect application of the upper shell (*n* = 2, 78/92 years, Senior).

**Table 2:** Summary of demographics per cohort

	Age [years]	Sex [% female]	Shoe size [EU]	VACO®ped [%Small]
Sport group	31 ± 7 (23–42)	27%	42 ± 2 (39–46)	36%
Cardio group	59 ± 11 (39–80)	58%	40 ± 2 (37–44)	58%
Senior group	82 ± 5 (76–92)	60%	41 ± 3 (38–46)	40%

### Application score

**Fig. 4:**
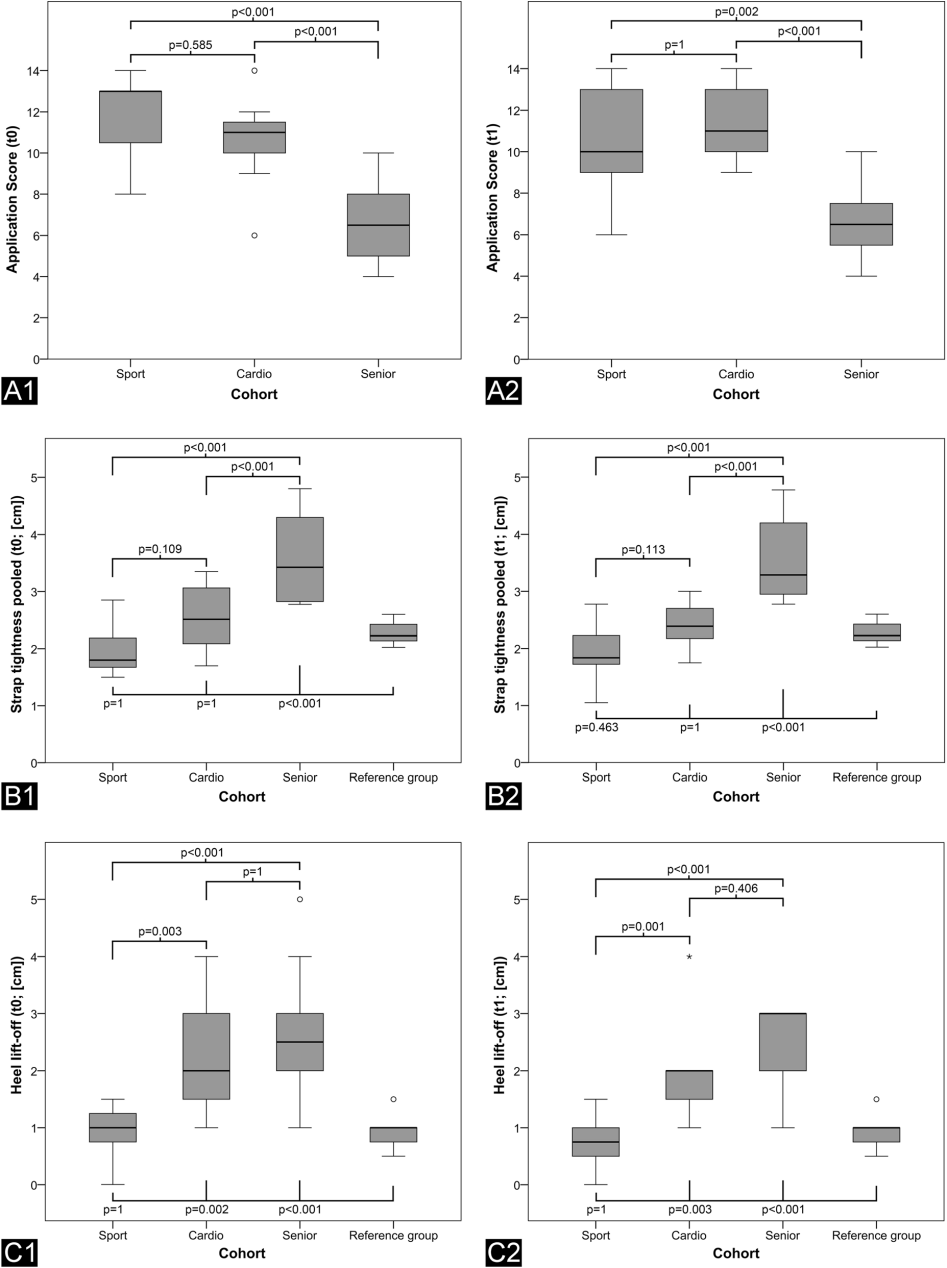
Boxplots illustration subgroup analysis of each variable per cohort. A: Application Score; B: Strap tightness pooled; C: Heel lift-off; 1: t0; 2: t1

The Application Score for all subjects taken together did not differ between t0 (9.9 ± 3 points) and t1 (9.8 ± 2.9 points; *p* = 0.508). Sex (*p* = 0.169 / *p* = 0.599) and VACO®ped size (*p* = 0.984 / *p* = 0.921) had no significant influence (t0 / t1). Age revealed a moderate correlation for the Application Score at t0 (*n* = 33; *R* = − 0.664; *p* < 0.001) but no significant correlation was observed at t1 with 4 drop-outs in the Senior group (*R* = − 0.309; *p* = 0.110). Analysing the Application Score per cohort revealed significantly inferior values for the Senior- (6.6 ± 2.2 / 6.6 ± 1.9) compared to the Cardio- (10.8 ± 2.1; *p* < 0.001 / 11.3 ± 1.8; *p* < 0.001) and Sport group (11.9 ± 1.9; *p* < 0.001 / 10.5 ± 2.7; *p* = 0.002), both at t0/t1 (Fig. 4A). No significant differences could be observed within each group when comparing t0 and t1 (Table 3). The application of the orthosis was insufficient (Application Score < 9) in 30% of the patients at t0 and in 34% at t1. 14% in the Sport-, 4% in the Cardio- and 83% in the Senior group achieved less than 9 points in the Application Score. The results of the Application Score for both time points are depicted for each group in in Table 3.

**Table 3:** Application Score per cohort for both time points (t0 and t1)

		t0	t1	*p*-value
Sport group	Application Score	11.9 ± 1.9 (8–14)	10.5 ± 2.7 (6–14)	0.122
	Application insufficient^a^	*n* = 1 (9%)	n = 2 (18%)	n.a.
Cardio group	Application Score	10.8 ± 2.1 (6–14)	11.3 ± 1.8 (9–14)	0.171
	Application insufficient^a^	n = 1 (8%)	*n* = 0 (0%)	n.a.
Senior group	Application Score	6.6 ± 2.2 (4–10)	6.6 ± 1.9 (4–10)	0.790
	Application insufficient^a^	*n* = 8 (80%)	*n* = 7 (88%)	n.a.

^a^Percentage of patients with an Application Score < 9 points

### Strap tightness

In the following, the data for strap tightness is presented for all four straps pooled. Strap tightness was significantly greater in the Reference group compared to all participants together at t0 (2.3 ± 0.5 cm vs. 2.7 ± 1.1 cm; *p* = 0.001) but not at t1 (2.3 ± 0.5 cm vs. 2.5 ± 0.9c m; *p* = 0.115). Volunteers’ strap tightness did not differ significantly between the two time-points of measurement (t0: 2.7 ± 1.1 cm; t1: 2.5 ± 0.9 cm; *p* = 0.445). Gender (t0: *p* = 0.178; t1: *p* = 0.107) had no significant influence on the results (t0: *p* = 0.906; t1: *p* = 0.466). A good correlation was found between age and strap tightness for t0 (*R* = 0.752; *p* < 0.001) and t1 (0.738; *p* < 0.001). Strap tightness showed significant differences between the different cohorts (*p* < 0.001). The Senior group (3.7 ± 0.8 cm / 3.6 ± 0.8 cm) had significant inferior results when compared to the Cardio- (2.5 ± 0.6 cm; *p* < 0.001 / 2.4 ± 0.4 cm; *p* < 0.001) and the Sport group (2.0 ± 0.9 cm; *p* < 0.001 / 1.9 ± 0.5 cm; *p* < 0.001), both at t0 / t1 (Fig. 4B). An additional file shows the data for strap tightness of each strap separately in more detail [see Additional file 1].

### Heel lift-off

Heel lift-off was significant less in the Reference- compared to the participants’ group at t0 (0.9 ± 0.3 cm vs. 2.0 ± 1.2 cm; *p* < 0.001) and t1 (0.9 ± 0.3 cm vs. 1.4 ± 0.9 cm; *p* = 0.009). There were no significant differences for heel lift-off between the volunteers at t0 and t1 (2.0 ± 1.2 cm vs. 1.4 ± 0.9 cm; *p* = 0.101). Gender (t0: 0.082; t1: 0.248) and VACO®ped size (t0: 0.420; t1: 0.351) did not significantly influence heel lift-off. Age showed a moderate correlation at both t0 (*R* = 0.632; *p* < 0.001) and at t1 (*R* = 0.661; *p* < 0.001). The Senior group again had the worst results (2.7 ± 1.2 cm / 2.5 ± 0.8 cm) but did not differ significantly to the Cardio group at t0/t1 (2.4 ± 1.1 cm; *p* = 1 / 1.9 ± 0.8 cm; *p* = 0.406). The Sport group (1.0 ± 0.5 cm / 0.8 ± 0.4 cm) showed significantly better results compared to the Cardio- (*p* = 0.003 / *p* = 0.001) and the Senior group (*p* < 0.001 / *p* < 0.001) at both t0 / t1 (Fig. 4C).

### Subjective judgement

Summarizing, 95% of the volunteers presumed the walker was applied correctly. In order to assess, whether the participants were able to subjectively judge if the orthosis was applied correctly, the subjective judgment was compared to the Application Score per its clinical effectiveness (Application Score values < 9 ≥ points). Overall, the application of the orthosis was insufficient (Application Score < 9 points) in 30% of the volunteers but only 5% subjectively judged the application incorrect. Consequently, out of 19 participants who applied the orthosis inefficient, 17 (90%) believed the application to be correct. Table 4 illustrates this relation per cohort.

**Table 4:** Application Score per subjective judgement per cohort

			Application insufficient^a^	Application sufficient^b^
Subjective judgement	Overall	Correct	17	42
		False	2	1
	Sport group	Correct	3	18
		False	0	0
	Cardio group	Correct	1	22
		False	0	1
	Senior group	Correct	13	2
		False	2	0

^a^Number of patients with an Application Score < 9 points

^b^Number of patients with an Application Score ≥ 9 points

## Discussion

Due to the advantage of removability, walker orthosis became increasingly popular. However, the walker must be re-applied by the patient himself. The application requires several tasks and demands physical strength to tighten the belt straps. Consequently, patients could face difficulties in self-handling the walker. The incorrect application could result in an insufficient immobilization of the ankle and therefore compromise the treatment outcome. Consequently, it is essential to ensure that patients can self-handle such an orthosis. To our best knowledge, no study has assessed the applicability of a walker. Of special interest were differences between cohorts of varying age and level of activity.

When interpreting the results of the Application Score, it is notable that the above outlined, standardized instructions resulted for both, the Sport- and the Cardio group, in comparably good results for the first (t0) testing. The Senior group, on the other hand, achieved inferior results at t0. Still, all participants had received the exact same standardized instructions. For our understanding, the favourable findings for the participants of the Sport- and Cardio group argue that the instructions provided were well understandable and reproducible. The application procedure of the VACO®ped showed a good reproducibility. None, the 8-point Application Score, strap tightness or heel mobility differed significantly between the two measurement occasions neither for all subjects taken together nor within each group individually. This does argue for the learning effect of the instruction session. Once the application of the orthosis was understood, the participants of the Sport- and the Cardio group were able to reproduce the procedure one week later. Visa versa, it appears reasonable, that those participants, who were unable to apply the orthosis directly after receiving the same standardized instructions, were also unable to apply the orthosis one week later. Taken together, as all participants received the same standardized instructions and the results differed significantly already at t0, this argues for a difference between the Sport- and Cardio group on the one hand and the Senior group on the other hand. That there is no difference between the two time points within each group seems comprehensible.

As outlined above, the handling of a walker orthosis requires residual memory capacity and physical strength. With increasing age and reduced level of activity, both, residual memory and strength, decline. The 8-step application of the walker requires cognitive ability. Up to 25% of people aged 70 years or above, suffer mild cognitive impairment (MCI) [11]. This means, their cognitive capacity is below the person’s expected age average. MCI-patients have greater difficulties and are less accurate in solving tasks of daily routine [12]. The prevalence of MCI is even greater among nursing home residents [13]. Although distinct cognitive impairment was considered an exclusion criterion in this study it remains a challenge to identify patients with MCI. Currently no single test is available but rather a set of criteria is required to diagnose MCI [12]. It can therefore be assumed, that the proportion of patients suffering MCI was considerably higher in the Senior- compared to the other groups. This would explain the significantly inferior results of the Senior Group for the Application Score (Fig. 4A). Two volunteers of the Senior group even forgot to apply the upper shell at the second measurement, which will definitely result in an insufficient immobilization.

The application of a walker also requires physical strength to tighten the belt straps. Impaired physical strength result in reduced strap tightness with a subsequently increased foot mobility and insufficient ankle immobilization. Up to 29% of community dwelling elderly suffer from sarcopenia [14]. Sarcopenia is defined as a progressive loss of skeletal muscle mass and strength that occurs with aging. It leads to physical disability and limited mobility. Again, institutionalized elderly are more frequently affected from sarcopenia compared to independently living elderly [15]. Therefore, it can be assumed that the proportion of patients suffering sarcopenia was the highest in the Senior-, compared to the other two groups. This would well explain the poor strap tightness and heel lift-off values achieved in the Senior Group (Fig. 4B/C). Moreover, it could explain exclusion of two seniors from retesting (t1) due to limited mobility.

Further, the participants were unable to correctly self-assess the proper application of the walker. Interestingly, most subjects considered the application of the walker to be correct. Even the majority of subjects with inferior results for the Application Score (< 9 points) (89%) considered the orthosis to be applied correctly. Furthermore, the two Senior participants who had to be excluded because they did not apply the upper shell correctly, considered the application to be correct. Therefore, the subjective perception of the subjects differed significantly from the parameters objectively assessed. Another important aspect is that the application of the walker orthosis was assessed in a healthy population. Medical conditions for which the orthosis is prescribed in patients might further hamper the use of the orthosis. Pain, reduced range of motion or concomitant injuries might affect the application. However, to answer this question was not the aim of the present study and will require investigation in future studies.

Taken together, the Senior group achieved inferior results in all outcome measures assessed. It must be hypothesized that the poor results for the Application Score, the inferior strap tightness in combination with the increased heel-rise does result in an insufficient immobilization of the ankle. This might have an impact on the treatment success. Consequently, it appears advisable to validate the proper application by the patient after giving instructions. Further, the appraisal of the patient cannot be relied on. Therefore, the treating physician should judge if the patient is capable to handle a walker. This could be done, for example, by having the patient put on the walker by himself while being supervised by the physician.

Several limitations of the study need to be discussed. First, the sample size of each group was rather small. Second, no detailed geriatric assessment, such as Barthel-Index or Mini-Mental-State Examination, was conducted. Furthermore, physical strength was not measured. These might have been helpful to further define the population at risk for mishandling a walker. Finally, retesting was limited to one occasion. In everyday life, patients most like remove and reapply the walker more often. Repetitive application could possibly result in a learning curve positively affecting the outcome parameters. Despite the above outline limitations, the study is inherent of several strengths. First, the volunteer-sample covered three cohorts with a broad age range and different levels of activity. Second, next to subjective outcome parameters, objective parameters were developed to objectify correct application and sufficient ankle immobilization. Finally, a reference group was introduced to correlate the results of the intervention groups.

## Conclusions

The present study revealed, that the cohort with the highest age and presumably the lowest level of activity was at risk for impaired handling of a walker orthosis. Moreover, it should be kept in mind, that subjective judgment by the patient seems to be misleading. The treating physician should therefore ensure the ability, especially of elderly patients, to apply the orthosis correctly in order to provide patient safety. Future studies should try to further characterize the population at risk for mishandling a walker orthosis especially in regard to discreet conditions such as mild cognitive impairment, sarcopenia reduced strength or similar. Furthermore, future studies should assess the applicability in patients with medical conditions.

## Additional file

Additional file 1: Detailed data for strap tightness**.** Shows the data for each strap separately at t0 and t1. (XLSX 37 kb)

## Abbreviations

ANOVA: Analysis of variance
MCI: Mild cognitive impairment
N: Newton
SD: Standard deviation
SPSS: Statistical Package for the Social Sciences
